# Preliminary evidence for a forestomach washing mechanism in llamas (*Lama glama*)

**DOI:** 10.1007/s42991-021-00142-1

**Published:** 2021-06-16

**Authors:** Jean-Michel Hatt, Daryl Codron, Henning Richter, Patrick R. Kircher, Jürgen Hummel, Marcus Clauss

**Affiliations:** 1 Clinic for Zoo Animals, Exotic Pets and Wildlife, Vetsuisse Faculty, University of Zurich, Winterthurerstr. 260, 8057 Zurich, Switzerland; 2 Department of Zoology and Entomology, University of the Free State, PO Box 339, Bloemfontein, 9300 South Africa; 3 Clinic for Diagnostic Imaging, Vetsuisse Faculty, University of Zurich, Winterthurerstr. 258c, 8057 Zurich, Switzerland; 4 Ruminant Nutrition, Department of Animal Sciences, University of Goettingen, Kellnerweg 6, 37077 Göttingen, Germany; 5 AgroVet Strickhof, Lindau Site, Eschikon 27, 8315 Lindau, Switzerland

**Keywords:** Camelid, Ruminant, Tooth wear, Chewing, Rumination, Grit, Phytoliths

## Abstract

Dust and grit are ingested by herbivores in their natural habitats along with the plants that represent their selected diet. Among the functions of the rumen, a washing of ingesta from adhering dust and grit has recently been demonstrated. The putative consequence is a less strenuous wear on ruminant teeth by external abrasives during rumination. The same function should theoretically apply to camelids, but has not been investigated so far. We fed six llamas (*Lama glama*) a diet of grass hay and a lucerne-based pelleted food in which fine sand had been included at about 8% of ingredients, for ad libitum consumption for 6 weeks. Subsequently, animals were slaughtered and content of the different sections of the gastrointestinal tract was sampled for the analysis of dry matter (DM), total ash, and acid detergent insoluble ash (ADIA, a measure for silica). Additionally, two of the animals were subjected to whole-body computer tomography (CT) after death in the natural sternal resting position. No clinical problems or macroscopic changes in the faeces were observed during the experimental period. The results indicate an accumulation of ADIA in the C3 compartment of the stomach complex, in particular in the posterior portion that is the equivalent of the abomasum in ruminants. By contrast, contents of the C1, from which material is recruited for regurgitation and rumination, were depleted of ADIA, indicating that the contents had largely been washed free of sand. The washing effect is an unavoidable side effect of the flotation- and sedimentation-based sorting mechanisms in the ruminant and the camelid forestomachs. In theory, this should allow ruminants and camelids to live in similar habitats as nonruminant herbivores at lower degrees of hypsodonty.

## Introduction

Many mammals inadvertently ingest relevant amounts of indigestible material with their natural diet, including dust, grit, sand and soil. For example, this is observed in insectivorous and especially myrmecophagous mammals (McNab 1984; Gull et al. 2015), but particularly in herbivores (Skipworth 1974; Arthur and Alldredge 1979; Arthur and Gates 1988; Beyer et al. 1994; Hummel et al. 2011; Turner et al. 2013; Sanson et al. 2017). Even though sand impaction is sporadically reported as a health problem in domestic animals, especially in horses (Hassel et al. 2020), the general perception is that the mammalian digestive tract can handle ingested soil quite well (Dirksen 2002; Husted et al. 2005; Kendall et al. 2008; Siwińska et al. 2019).

A typical adaptation against the abrasiveness of ingested soil are hypsodont (high-crowned) teeth, which occur particularly in herbivores in arid environments (Damuth and Janis 2011; Jardine et al. 2012). The observation that extant ruminants do not achieve the same degree of hypsodonty as equids (Kaiser et al. 2013) has led to the theory that the ruminant forestomach washes off abrasives from the digesta before it is regurgitated for thorough mastication in the rumination process (Semprebon et al. 2019), and this washing mechanism has recently been demonstrated in goat and sheep (Hatt et al. 2019, 2020). Due to a series of similarities between ruminants and camelids, a similar washing mechanism is expected in the latter.

**Fig. 1:**
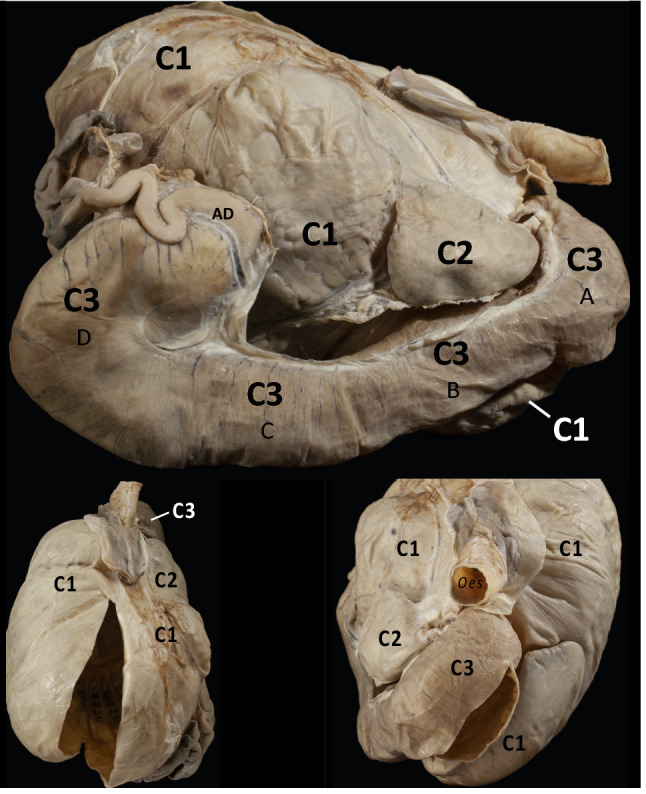
Photographs of a llama (*Lama glama*) stomach complex fixated in situ (with both the C1 and the C3 opened for access to the inside). Top: lateral right view (oesophagus pointing towards the right side of the picture), corresponding to the drawing in Fig. 2. Bottom left: dorsal view (oesophagus pointing towards the top of the picture). Bottom right: frontal view (oesophagus opening pointing towards the viewer). Note the relationships of the C2 to both the C1 and the C3 compartments. The C3 is located cranially, ventrally and caudally to the C2. The specimen did not originate from the present study but had been produced earlier by Urs Müller, preparator at the Institute of Veterainary Anatomy, Vetsuisse Faculty, University of Zurich

The camelid forestomach is generally divided into three macroscopically distinct compartments (Fig. 1). Although some authors use the same terminology as in ruminants to describe these, this is not supported unanimously (reviewed by Langer 1988). Following Vallenas et al. (1971), they are referred to as the voluminous C1 (the first compartment, the functional equivalent of the rumen), a small C2 (the second compartment, the functional equivalent of the reticulum), and a tubular C3 (the third compartment). The proximal parts of the C3 (parts A–C in Fig. 1), also referred to as the ‘gastric tube’, are functionally similar to the ruminant omasum, but anatomically very different. The last part of the C3 (part D in Fig. 1) is lined by a glandular epithelium that corresponds to that of the abomasum. The camelid forestomach contains so-called glandular sacs in some areas of its C1 (Fig. 1). The openings between the C1 and C2, and between the C2 and C3, are of smaller magnitudes than the openings between rumen and reticulum, or between reticulum and omasum, in ruminants of similar body size (Pérez et al. 2016), which might be linked to the generally lower food intake in camelids (Dittmann et al. 2014).

Apart from the fermentation of plant material by microbes with the corresponding production of volatile fatty acids in the C1 (Vallenas and Stevens 1971), the camelid forestomach has analogous physiological characteristics to that of the ruminants: the contents of the rumen or C1, respectively, are stratified, as evidenced by moisture and particle size distribution (Clauss et al. 2009; Idalan et al. 2019) or by computer tomography (CT) (Van Hoogmoed et al. 1998; Braun et al. 2011; Stieger-Vanegas and Cebra 2013); the reticulum or C2 contains particularly moist contents; the omasum or proximal C3 remove water from the digesta; and beyond the reticulum or C3, the digesta does not contain large particles (Lechner-Doll and von Engelhardt 1989; Clauss et al. 2017; Idalan et al. 2019). Large particles are selectively retained by the forestomach in both taxonomic groups compared to small particles (Lechner-Doll et al. 1990; Dittmann et al. 2015), based on a density-dependent sorting mechanism (Lechner-Doll et al. 1991). This ensures that large particles are selectively re-submitted to rumination (Hendrichs 1965; Dittmann et al. 2017), leading to particularly fine faecal particles in both ruminants and camelids as compared to other similar-sized herbivores (Fritz et al. 2009; Clauss et al. 2015). During ingestion, both ruminants and camelids show a less thorough mastication pattern, in contrast to their respective rumination cycles (Dittmann et al. 2017), possibly to delay thorough mastication until after the digesta has been washed. Finally, extant representatives of both ruminants and camelids do not display the high-crowned teeth observed in equids (Kaiser et al. 2013), even though especially the camelids are associated with arid habitats.

**Fig. 2:**
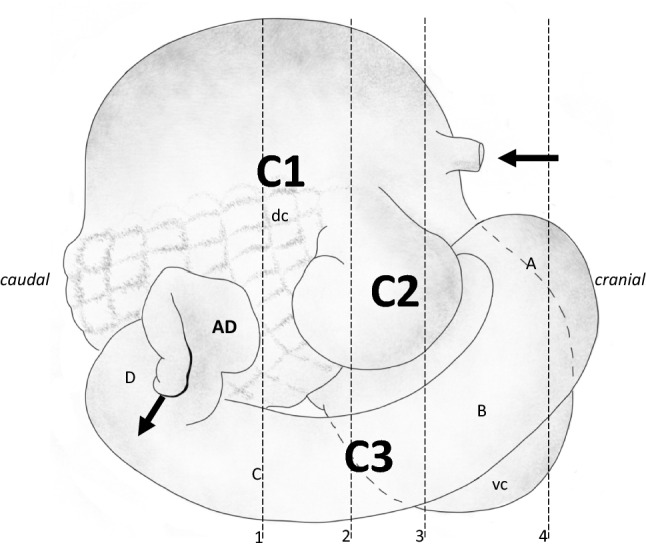
Schematic representation of the camelid forestomach, viewed from its right side. The cranial part is to the right, with the oesophagus (arrow), and the caudal part to the left. While the forestomach is positioned next to the left abdominal wall of the animal, the intestines are placed behind it and on its right side in the abdominal cavity, between the the C1 and C3, and above the C3, and are not shown here. Between the viewer and the front part of the stomach complex, a part of the liver would be situated as well. C1 first compartment (with a ‘dc’ dorsocaudal part from where contents are regurgitated for rumination) and a ‘vc’ ventrocranial part (partly overlaid by the C2 and C3); note that both parts of the C1 have areas of glandular sacs on the inside that are also visible from the outside for the dc; C2 second compartment; C3 third compartment with three sections (A-C) without glandular epithelium and a final section (D) with glandular epithelium, which opens into the Ampulla duodeni (AD), the first part of the duodenum. Drawing modified from Vallenas et al. (1971). After being swallowed, digesta typically moves a variable number of times between the dorsal and ventral C1, and then into the C2. Dense material (typically, small particles) passes from the C2 to the C3, whereas lighter material (typically, large particles) is propelled backwards from the C2 towards the C1. Material from the dorsal C1 is regurgitated for rumination. The numbered dashed lines correspond to the transects respresented by the four CT images in Fig. 3A

The present study was conducted to assess whether a similar washing mechanism as in ruminants (Hatt et al. 2019, 2020) could be demonstrated in camelids. One of the methods used was computed tomography (CT). Because previous descriptions of the camelid forestomach with CT images (Van Hoogmoed et al. 1998; Stieger-Vanegas and Cebra 2013) deviate in their identification of the C2 from anatomical displays of fixated specimens (Fig. 1), photographs (Pérez et al. 2016) or schematic drawings (Vallenas et al. 1971; Fig. 2), special attention was directed towards the identification of the C2 in CT images.

## Methods

Experiments were performed with approval of the Swiss Cantonal Animal Care and Use Committee Zurich (animal experiment licence 003/2019). Six mature (older than 5 years), non-reproducing llamas (one intact male, one castrated male, four intact, non-pregnant and non-lactating females) from private llama breeders, designated for slaughter, were kept at a common facility for 6 weeks prior to the designated slaughter date. During this time, they were kept as a group, on a diet of grass hay, pellets, and access to a grass pasture. The condition of the animals and the visual appearance of the faeces were controlled on a daily basis.

The pellet had a base of lucerne meal, which is naturally low in phytoliths. External abrasives (SCR-Sibelco N.V., Antwerp, Belgium) were manufactured into the pellets in the form of silica as fine sand (METTET AF100, mean particle size of 130 µm, representing ‘grit’) at a concentration of 8% of all pellet ingredients; the same diet had been amongst those used to assess the washing mechanism in sheep (Hatt et al. 2020). The pelleted diet and the grass hay were offered for ad libitum consumption. It was not possible to measure food intake on an individual basis. The nutrient composition of pellets and hay is given in Table 1.

**Table 1:** Nutrient composition of the hay and pelleted diet fed to llama (*Lama glama*) in the present study

Analyte	Grass hay	Pelleted diet
Total ash	82	202
Crude protein	80	149
Crude lipids	12	24
Acid detergent fibre	393	297
Acid detergent insoluble ash (silica)	24	100

Values in g/kg dry matter

At the end of the 6 weeks, animals were slaughtered by bolt stunning and exsanguination at two different slaughtering facilities. Whereas four animals were designated for human consumption and had to be processed immediately after death, two animals were designated as animal food for a wildlife park, and could be subjected to CT directly after death, placed in the natural sternal resting position. CT images were acquired using a helical multi-slice scanner (Siemens Somatom Sensation Open with sliding gantry, Siemens Medical Solutions, Erlangen, Germany) to image the abdomen of the animal (tube voltage at 120 kVp, image matrix of 512 × 512 pixels, field of view of 1329 × 762 pixels, slice thickness of 0.6 mm, B30s convolution kernel).

CT data sets were converted to DICOM medical imaging format and evaluated in Horos v3.3.6 (Horos Project 2019). Radiodense silica volumes (cm^3^) were calculated by manually defining regions of interest (ROIs) on every sixth slice and automated interpolation of missing ROIs. To guide the interpretation of the CT images, please refer to the fixated specimen in Fig. 1 and the schematic visualisation of the camelid stomach in Fig. 2. Note that the specimen from which the fixated stomach was produced was not part of the present study, but an approximately 8-year-old female llama euthanized for medical reasons unrelated to the gastrointestinal tract several years earlier.

After death or CT, the six carcasses were opened and the gastrointestinal tract (GIT) was exenterated, taking care of not kneading or turning its sections, to the effect that the contents of the different sections remained representative for these sections. Subsequently, samples were taken from the dorsal C1 (from where material is regurgitated for rumination), ventral C1, C2 (sorting forestomach), the proximal C3 (fluid reabsorption forestomach) in three sections (A–C), the distal C3 D (stomach, initiation of auto-enzymatic digestion), small intestine, caecum, proximal colon, spiral colon, and rectum (faeces). Standard nutrient analyses (AOAC 1995) were applied. Samples were analysed for the concentration of dry matter (dried at 103 °C) and total ash (i.e., including not only silica but also minerals; AOAC no. 942.05), for analyses for acid detergent fibre (ADFom, AOAC no. 973.18) and acid detergent insoluble ash (ADIA) as a proxy for silica (Hummel et al., 2011).

Differences across gastrointestinal tract sections were assessed using Mixed Effects Linear Models in R 3.4.3 (R_Core_Team, 2015), incorporating individual as a random factor. Each variable [dry matter, silica (ADIA), total ash] was tested with Tukey’s HSD post hoc test for multiple comparisons (significance level at 0.05).

## Results

Throughout the 6 weeks, all animals were observed daily to ingest the pelleted diet, to feed on grass hay, and to graze. The estimated amount of pellets consumed by the whole group was between 6 and 12 kg per day. During the whole time, no animal showed clinical signs corresponding to sand impaction, such as reduced appetite, lack of defecations, or a body posture indicative of abdominal pain. All animals defecated normally throughout the study, and the faeces appeared normal at visual inspection.

**Fig. 3:**
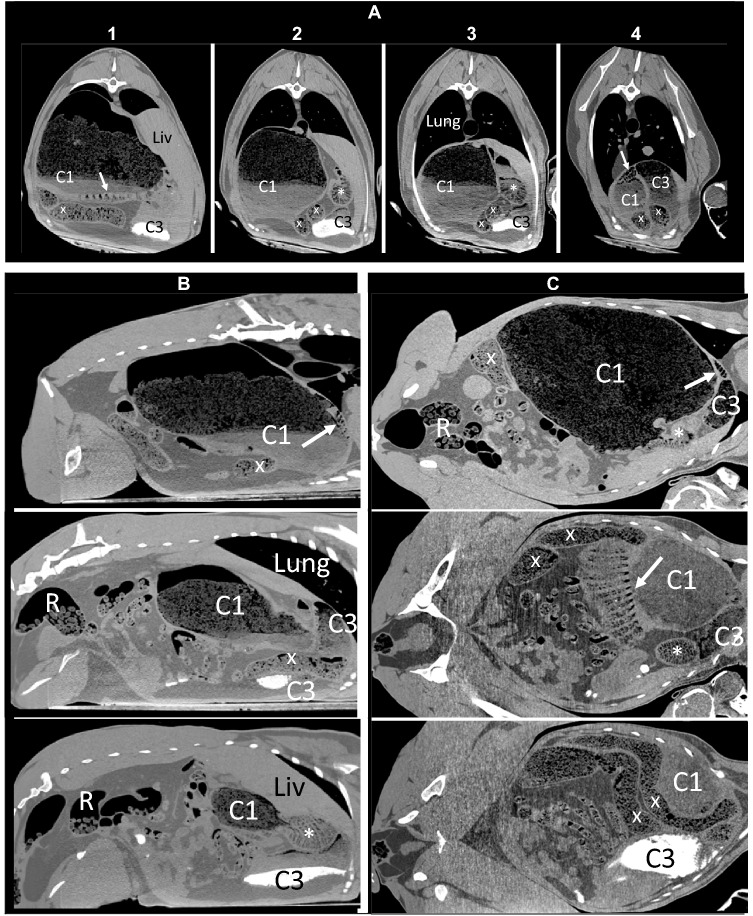
Computed tomography images of thorax and abdomen of a llama (*Lama glama*) fed a pelleted diet that included sand and grass hay for six weeks. Images represent **A** vertical slices in caudo-cranial view. The numbering corresponds to the dashed lines in Fig. 2, with the most cranial image on the right and the most caudal on the left; **B** sagittal slices in view from the right side, from the left (top) to the right part (bottom); **C** horizontal slices in dorso-ventral view, from the dorsal (top) to the bottom part (bottom). Note the dorso-ventral contents stratification in the C1 (with air, particulate matter with gas inclusions, and a fluid layer) and the glandular sacs (indicated by arrows). The C2 lies on the right side in the middle of the C1 (indicated by *) with its reticulated structure clearly visible. The C3 is cranial and ventral to the C2, and contains radiodense material in its ventral portion. Colon indicated by x, rectum filled with formed faeces by R. Liv = liver

The CT images showed the typical camelid anatomy (Fig. 3), including the glandular sacs of the C1 and the honeycomb-like inside structure of the C2 (marked in Fig. 3 by *). The C3 originates cranially from the C2 (Fig. 3B, C) and then passes ventrally to the C2 towards the back (Fig. 3B). A stratification of the C1 contents was evident, with a gas layer on top of a fibre layer characterized by gas inclusions, which was again on top of a liquid (Fig. 3).

In both animals in which a CT could be made, radiodense material was visible in the C3 (Fig. 3). This amounted to 12 cm^3^ in the animal whose C3 contents had a silica (ADIA) concentration of 10% in dry matter, but to 245 cm^3^ in the animal where this was up to 90% in dry matter. In the latter animal, sand was also visible at various locations in the small intestine. In the former animal, some individual glandular sacs of the C1 were filled with radiodense material.

**Fig. 4:**
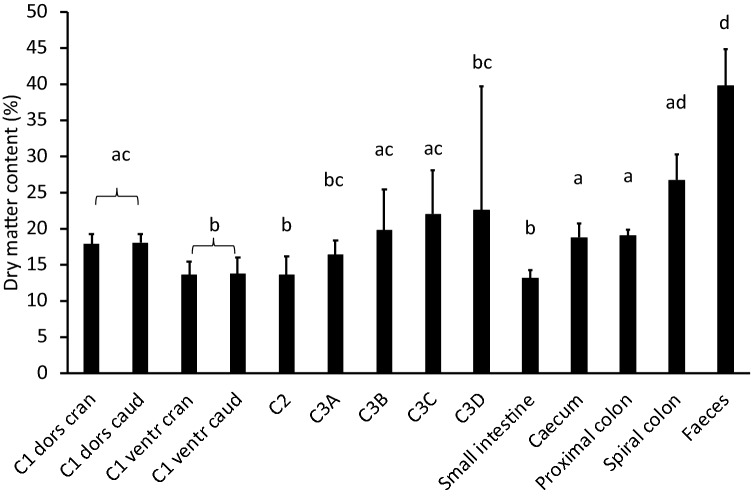
The mean (+ SD) dry matter concentration in the contents of different gastrointestinal tract sections in six llamas (*Lama glama*) fed grass hay and a pellet of high abrasive content. Note the lower dry matter (= higher moisture) in the ventral C1 due to digesta stratification and the C2, and the increase in dry matter from C3A–C3C. The unexpectedly high dry matter concentration in the C3D is suggestive of sand accumulation. From the small intestine to the rectum, moisture is continuously absorbed, leading to comparatively dry faeces. Columns not sharing a letter are significantly different

**Fig. 5:**
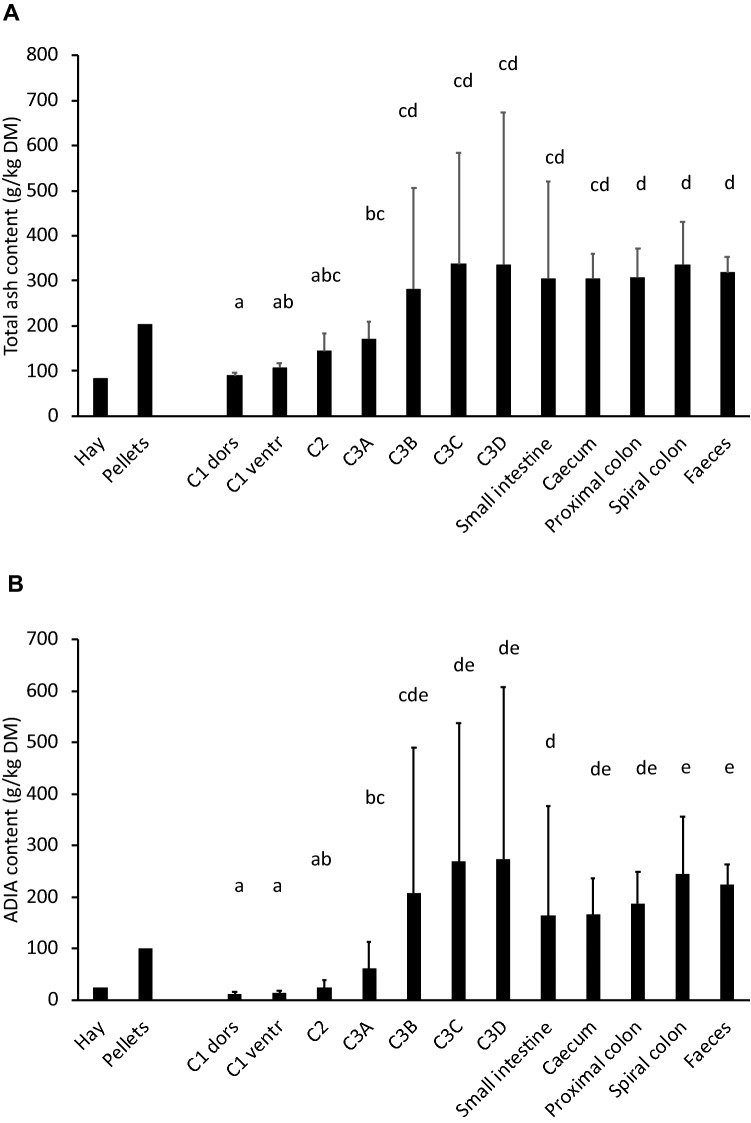
The mean (+ SD) concentration of **A** total ash (including macro-minerals and trace minerals), **B** acid detergent insoluble ash (ADIA; a measure for silica) in the diet and digestive tract of six llamas (*Lama glama*) fed grass hay and a pellet of high abrasive content. Note the depletion of ash and ADIA in the C1 and their accumulation in the C3. Columns representing different gastrointestinal tract sections not sharing a letter are significantly different

The dry matter concentration in the contents of the different gastrointestinal sections followed the expected pattern of a decrease in the ventral C1 and the C2, a gradual increase along the C3, and an increase from the small intestine to the rectum (Fig. 4). The only deviation from the expected pattern was that the distal part of the C3 (C3D) did not show a decrease in dry matter, but rather an increase, indicative of sand at this location. Concentrations of total ash and of ADIA (silica) were both lower in the C1 than in the pelleted diet, and increased from C2 to a peak at the two last sections of the C3 (Fig. 5). Silica concentrations dropped again in the small intestine, caecum and proximal colon, and increased towards in the spiral colon. The faeces contained 223 ± 41 g silica/kg dry matter (Fig. 5).

## Discussion

The present study underlines the functional similarities between the ruminant and camelid digestive tracts and reveals preliminary evidence for the presence of a forestomach washing mechanism in camelids. We consider this a pilot study, because the results are based on only six animals; because food intake could not be controlled completely, with animals ingesting unknown proportions of sand-containing pelleted food, grass hay, and pasture grass; and because the period the animals were under observation was comparatively short at 6 weeks.

Similar to the findings in ruminants (Hatt et al. 2019, 2020), no clinical problems were evident in the llamas, in spite of the evident sand accumulation in the C3. There are only very sparse reports on sand accumulation in camelids and related clinical problems. Tharwat (2020) describes sand in the ‘rumen’ of a dromedary (*Camelus dromedarius*) after excessive sand ingestion, but the image provided could as well show the tubular C3. Abutarbush and Petrie (2006) reported the accumulation of sand in the C3 and in the spiral colon of a 1-month-old alpaca (*Vicugna pacos*) with fatal consequence; several other animals of the same herd had sand in their faeces but did not show clinical signs. Surveys on problems of the digestive tract of camelids do not mention sand impaction (Cebra et al. 1998; Theuß et al. 2014). Given the high likelihood that camelids inadvertently ingest dust and grit when feeding in their natural habitats, it appears plausible that their digestive tract can routinely handle these substances. The observation that sand is not excreted continuously at the same rate that it is ingested, but with a delay after accumulating, to some degree, in the C3 of camelids or the abomasum of ruminants (Hatt et al. 2019, 2020) is most likely due to the voluminous cavity represented by these organs, their ventral position in the abdominal cavity that reminds of a household odor-trap siphon, and the fact that their exit into the lower intestinal tract represents a clear muscular barrier with a decrease in diameter. Once a certain degree of fill by sand is reached at this location, we hypothesise that the excretion rate of sand begins to correspond to its ingestion rate.

The camelid forestomach was previously depicted by CT by Van Hoogmoed et al. (1998) and Stieger-Vanegas and Cebra (2013). In both studies, the label ‘C2’ was allocated to a structure that was most cranial, and in the case of the latter study, even on the left side of the stomach complex (Fig. 3 of the former and Fig. 1C of the latter publication). To our opinion, that structure corresponds to the cranial C3 or the ventral C1; the C2, by contrast, is located on the right side of the camelid stomach complex, and, in contrast to its analogue, the ruminants’ reticulum, is not the most cranial structure of the complex (Figs. 1, 2, 3). In these studies, contrast materials were applied to the animals. This reduced the details including the reticulated pattern of the C2 and might have resulted in erroneous assignment.

The fact that the camelid forestomach contents are stratified, similar to that observed in cattle-type ruminants, has been confirmed previously using CT images and content analyses (see “Introduction”), and was again demonstrated by both methods in the present study. These findings support the general interpretation of the mechanical processes occurring in the camelid forestomach (see “Introduction”).

For ruminants, one of the hypothesized consequences of the forestomach washing mechanism is that they did not have to evolve the same degree of hypsodonty as other similar-sized but simple-stomached grazers, namely the equids, and based on our results, we expect the same for camelids. Although camelid hypsodonty increased over fossil time, the extant camelids are among the extant ruminants in their hypsodonty index (Semprebon and Rivals 2010), supporting this notion. Extant camelids may be constrained in their natural distribution to resource-poor (and typically arid) habitats due to lower intake and metabolism compared to ruminants (Dittmann et al. 2014) that have been hypothetically linked to the anatomy of the camelid forestomach (Pérez et al. 2016). The washing mechanism ensures that the putatively high loads of sand and grit in these habitats are not problematic.

## Conclusion

In conclusion, analyses of the content of the digestive tract in llamas fed sand-containing pellets indicated that the first and largest forestomach compartment, the C1, was depleted in sand in comparison to the food, suggesting a washing mechanism that removes sand, possibly prior to rumination. Content analyses as well as CT images of the digestive tract indicate that the sand accumulates in the C3 as it does in ruminants in the abomasum, probably due to the similarity in shape and anatomical position. Reducing the abrasive load on teeth by washing off grit prior to rumination is an important effect of the ruminant and the camelid forestomach.

## Data Availability

The original data are available from the authors upon reasonable request.
